# Integrative regulation of cerebral blood flow in response to exercise and environmental extremes

**DOI:** 10.1113/EP093853

**Published:** 2026-05-19

**Authors:** Shigehiko Ogoh, Damian M. Bailey

**Affiliations:** ^1^ Department of Biomedical Engineering Toyo University Saitama Japan; ^2^ University of South Wales Pontypridd UK; ^3^ Bexorg Inc New Haven Connecticut USA

The concepts of ‛Exercise is Medicine®’ and ‛Exercise as Medicine’ both highlight the therapeutic role of physical activity in maintaining health. Specifically, ‛Exercise is Medicine®’ denotes a global health initiative led by the American College of Sports Medicine and the American Medical Association, focused on promoting physical activity within clinical care (Sallis, 2009). In contrast, ‛Exercise as Medicine’ is more appropriately used as a research framework where exercise is conceptualized as a therapeutic intervention for established diseases (Febbraio & Pedersen, 2026). Although these terms differ conceptually, both emphasize that regular exercise is vital for health; in support, it is recommended that healthy adults perform at least 150 min of moderate‐intensity dynamic exercise per week. A substantial body of evidence indicates that regular exercise reduces the risk of cognitive decline and dementia, particularly in older adults (Burley et al., 2016; Calverley et al., 2020; Gomez‐Pinilla & Hillman, 2013; Mandolesi et al., 2018). Notably, chronic exercise has been shown to increase hippocampal volume (Erickson et al., 2011), suggesting that it may counteract age‐related brain atrophy. Ageing is associated with reductions in synaptic plasticity, decreased neurogenesis, brain volume loss, impaired cerebral blood flow (CBF) regulation, and attenuated neurovascular coupling (Matura et al., 2017; Tyndall et al., 2018). Chronic exercise appears to mitigate these deleterious changes by promoting both neural and vascular remodelling. These benefits include enhanced neurogenesis, synaptogenesis and gliogenesis (within hippocampus and neocortex), increased neurotrophic factors (e.g., brain‐derived neurotrophic factor, insulin‐like growth factor 1), angiogenesis (hippocampus, neocortex and cerebellum), modulation in neurotransmission systems (e.g., serotonin, noradrenalin and acetylcholine) and increased cerebral blood flow (Mandolesi et al., 2018). Collectively, these exercise‐induced structural and physiological adaptations in the brain may enhance cognitive reserve and resilience to neurodegeneration. In contrast, acute exercise induces transient physiological changes, such as temporary increases in CBF, alterations in neurotransmitter activity and short‐term improvements in cognitive performance (Ogoh et al., 2014). However, increases in global CBF are not consistently associated with cognitive enhancement (Ogoh et al., 2024), suggesting that acute exercise‐related cognitive benefits cannot be explained solely by elevated cerebral perfusion. Instead, transient neurochemical modulation, region‐specific perfusion changes and improved neural efficiency may play more critical roles. Thus, acute exercise primarily induces functional modulation, whereas chronic exercise leads to structural reorganization of neural and vascular systems. The long‐term cognitive benefits of exercise are therefore likely mediated not simply by increased CBF, but by qualitative improvements in neurovascular coupling and cerebrovascular regulation. Against this background, cerebral physiological adaptations appear to be crucial for maintaining brain health; however, the underlying mechanisms remain poorly understood. Accordingly, research focusing on the cerebral physiological mechanisms underlying exercise is essential for developing effective strategies to preserve brain function, particularly in older adults.

In recent years, rapid technological advances have accelerated research on the brain, an organ that still retains many unresolved and enigmatic aspects. Exercise science is no exception to this trend, with growing interest in exercise‐induced changes in cerebral circulation, autonomic regulation and cognitive function. From the perspective of cerebral circulation, research involving exercise increased markedly following the publication of a seminal paper that employed transcranial Doppler ultrasound (TCD) in 1982 (Aaslid et al., 1982). A PubMed search using the keywords ‛cerebral blood flow’ and ‛exercise’ identifies only three relevant articles published before 1982, whereas 2110 papers have been published since then, underscoring the profound impact of this methodological innovation on this research field. Notably, the number of publications has increased exponentially since around 2000. This expansion has been driven largely by methodological advancements, including the development of Doppler‐based techniques such as duplex ultrasound and analytical approaches like transfer function analysis, alongside the integration of near‐infrared spectroscopy and functional magnetic resonance imaging.

It is now widely accepted that CBF increases during exercise, at least at low to moderate intensities, which are defined as exercise performed near the first ventilatory threshold (VT1), although reductions may occur during high‐intensity exercise performed above the second ventilatory threshold (VT2), also referred to as the respiratory compensation point. However, until the early 1990s, the prevailing view was that CBF is tightly regulated and remains essentially constant during exercise. This concept was grounded in the best available evidence at the time and was strongly influenced by integrative models of circulatory regulation. In particular, a book entitled *Human Circulation: Regulation During Physical Stress* by the late Loring B. Rowell (1986), is widely regarded as a foundational text in circulatory physiology and presented schematic representations of cardiac output redistribution during exercise. These figures depicted reductions in visceral blood flow and marked increases in skeletal muscle perfusion, while blood flow to the kidneys and brain was assumed to remain unchanged during exercise. This framework became broadly accepted within the research community. Nevertheless, direct measurements of CBF velocity using TCD during exercise consistently demonstrated clear increases in cerebral perfusion. Similar observations had already been reported in earlier TCD studies, yet the discrepancy between these empirical findings and the entrenched notion of invariant CBF during exercise received surprisingly little critical discussion.

Many earlier studies (Globus et al., 1983; Madsen et al., 1993; Scheinberg et al., 1954) supporting the constancy of CBF relied on the Kety–Schmidt method (Kety & Schmidt, 1946). However, a portion of the inhaled isotope enters the circulation of scalp vessels, leading to an underestimation of global CBF compared with the true cerebral value (Obrist et al., 1967). Importantly, tracer‐based techniques used to estimate global perfusion reflect physiological constructs that differ fundamentally from those assessed by regional cerebral perfusion methods, such as TCD. It was subsequently hypothesized that exercise‐induced alterations in venous drainage could influence tracer kinetics and thereby bias CBF estimates obtained with this method (Ogoh & Ainslie, 2009a). This concern was later supported by experimental evidence demonstrating that, during exercise, internal jugular venous blood flow decreases, whereas vertebral venous outflow increases (Sato et al., 2017). Because the Kety–Schmidt method assumes equivalence between arterial inflow and venous outflow, this fundamental assumption is violated during exercise. Consequently, exercise‐induced increases in CBF are underestimated. This example highlights how methodological limitations can profoundly shape – and potentially mislead – physiological interpretation.

In our previous study (Bailey et al., 2011), we reported that acute exercise can sometimes ‛over‐challenge’ the brain, with molecular evidence of blood–brain barrier (BBB) disruption following autoregulatory breakthrough. This is important for understanding the beneficial effects of exercise intensity on CBF regulation. However, a similar issue to that discussed above regarding CBF during exercise also arises in the long‐standing concept of cerebral autoregulation. In the early development of this concept, which is crucial for understanding CBF regulation, methodological limitations were present, and the characteristics were not accurately described.

Cerebral vessels, like renal vessels, have traditionally been believed to possess a unique autoregulatory capacity that stabilizes CBF across a wide range of arterial pressures. Since the late 1990s (Blaber et al., 1997; Zhang et al., 1998), the application of transfer function analysis has enabled relatively straightforward assessment of dynamic cerebral autoregulation, leading to a marked increase in publications in this field. The intellectual foundation of cerebral autoregulation can be traced back to Lassen's landmark paper published in 1959 (Lassen, 1959), from which the well‐known ‛Lassen autoregulatory curve’ was derived – a figure that remains ubiquitous in physiology textbooks. It is noteworthy that Lassen's original representation appears to mirror earlier observations by Shipley & Study (1951), suggesting a longer historical evolution of this concept than is often recognized. While Lassen's model became the standard, its inherent limitations were later addressed by Heistad and Kontos (1983), who challenged the notion of a perfectly horizontal plateau by proposing a non‐zero slope. Crucially, the striking aspect was not the curve itself, but the manner in which it had been derived, as highlighted prior (Ogoh & Ainslie, 2009a). Lassen's curve was constructed by plotting resting global CBF determined by the Kety–Schmidt method against resting arterial blood pressure (ABP) across different participants consisted of normotensive, hypotensive and hypertensive individual patients (Lassen, 1959). Consequently, substantial inter‐individual variability, along with multiple confounding physiological factors, was inherently included in this relationship. Strictly speaking, valid assessment of autoregulation required within‐participant manipulation of ABP, rather than between‐participant comparisons.

That such a curve was accepted for nearly half a century as definitive evidence of a broad autoregulatory plateau, where CBF was assumed to remain constant across a blood pressure range of 60–150 mmHg (i.e., a plateau exceeding 100 mmHg) (Lassen, 1959), is deeply unsettling. This interpretation implied that cerebral perfusion would be maintained over a remarkably wide range of arterial pressures, far exceeding the magnitude of spontaneous blood pressure fluctuations encountered in daily life. Motivated by this concern, a subsequent study systematically manipulated ABP within the same individuals and quantified CBF responses using the TCD technique. Although TCD assesses regional blood velocity rather than global CBF, unlike Lassen's original data, this approach enabled identification of the ‘true’ autoregulatory curve. The results demonstrated that CBF and cerebral oxygenation are not independent of changes in blood pressure and that the plateau region appears to be substantially narrower than previously assumed. These findings indicate that a paradigm shift in the concept of cerebral autoregulation is required (Lucas et al., 2010). This history underscores a broader lesson: advances in measurement technology do not merely refine existing concepts, but can fundamentally challenge long‐held assumptions.

CBF is regulated not only by intrinsic cerebrovascular mechanisms but also by a wide array of interacting physiological systems, including the systemic circulation, arterial blood gases, respiratory control, autonomic function, intracranial pressure, arterial blood pressure regulation and redox‐related signalling mediated by reactive oxygen and nitrogen species (Bailey et al., 2007, 2017, 2018; Davison et al., 2002; Fogarty et al., 2011; Ogoh, 2008, 2017, 2019, 2025; Ogoh & Ainslie, 2009a, 2009b; Ogoh & Tarumi, 2019). The integration of these mechanisms renders CBF regulation highly complex and dynamic, making it inherently difficult to dissociate cerebral haemodynamics from brain function. This complexity underscores the need for integrative approaches in brain research. Although studies of CBF–brain function interactions have expanded rapidly with conceptual and methodological advances, re‐evaluation of long‐standing concepts has revealed that some widely accepted interpretations were shaped by methodological limitations. Importantly, revisiting these concepts using contemporary techniques represents not a critique of earlier work, but a natural progression of scientific understanding, through which established models can be refined or revised.

Environmental extremes, such as hypoxia and heat stress, profoundly challenge cerebral circulation and brain function by simultaneously perturbing cardiovascular, respiratory, metabolic and neural control systems, much like exercise itself (Ando et al., 2023; Ogoh et al., 2013; Ogoh et al., 2022; Shibasaki et al., 2020; Stacey et al., 2025). Rather than confounding factors, these stressors offer unique experimental opportunities to reveal the integrative and adaptive limits of CBF regulation. Understanding how exercise‐ and environment‐induced alterations in cerebral haemodynamics influence brain function, including cognition, has the potential to reshape current models of physiological regulaton and to uncover mechanisms of vulnerability in both health and disease. This special issue of *Experimental Physiology* brings together a diverse set of reviews covering cFMD methodologies, cerebral autoregulation, diet–cognition interactions, vascular properties and arterial stiffness, exercise interventions in head injury, brain metabolism, and cerebral circulation responses under heat and hypoxic stress. In this editorial, we note that some existing studies contain misconceptions or unverified findings; nevertheless, these cumulative efforts are crucial for advancing our understanding of the brain's largely unexplored physiological functions. Moreover, the brain is an organ highly susceptible to diverse influences, underscoring the importance of considering its complexity.

Consequently, research on CBF regulation under exercise and environmental stress should be viewed not as a niche extension of exercise physiology, but as a critical framework for advancing a more unified and mechanistic understanding of brain function. By presenting these findings in a unified forum, this special issue contributes significantly to deepening our understanding of the relationship between exercise and the brain, and to promoting research aimed at maintaining and improving brain health and cognitive function.

## AUTHOR CONTRIBUTIONS

Shigehiko Ogoh and Damian M. Bailey conceived and designed this Editorial. Shigehiko Ogoh conceptualized the work and wrote the first draft of the manuscript. Damian M. Bailey supervised the project and provided critical revisions to the manuscript. Both authors have read and approved the final version of this manuscript and agree to be accountable for all aspects of the work in ensuring that questions related to the accuracy or integrity of any part of the work are appropriately investigated and resolved. All persons designated as authors quality for authorship are listed.

## CONFLICT OF INTEREST

The authors have no conflicts of interest to declare that are relevant to the content of this article.

## References

[eph70314-bib-0001] Aaslid, R. , Markwalder, T. M. , & Nornes, H. (1982). Noninvasive transcranial Doppler ultrasound recording of flow velocity in basal cerebral arteries. Journal of Neurosurgery, 57(6), 769–774. 7143059 10.3171/jns.1982.57.6.0769

[eph70314-bib-0002] Ando, S. , Tsukamoto, H. , Stacey, B. S. , Washio, T. , Owens, T. S. , Calverley, T. A. , Fall, L. , Marley, C. J. , Iannetelli, A. , Hashimoto, T. , Ogoh, S. , & Bailey, D. M. (2023). Acute hypoxia impairs posterior cerebral bioenergetics and memory in man. Experimental Physiology, 108(12), 1516–1530. 37898979 10.1113/EP091245 PMC10988469

[eph70314-bib-0003] Bailey, D. M. , Evans, K. A. , McEneny, J. , Young, I. S. , Hullin, D. A. , James, P. E. , Ogoh, S. , Ainslie, P. N. , Lucchesi, C. , Rockenbauer, A. , Culcasi, M. , & Pietri, S. (2011). Exercise‐induced oxidative‐nitrosative stress is associated with impaired dynamic cerebral autoregulation and blood‐brain barrier leakage. Experimental Physiology, 96(11), 1196–1207. 21841038 10.1113/expphysiol.2011.060178

[eph70314-bib-0004] Bailey, D. M. , Lawrenson, L. , McEneny, J. , Young, I. S. , James, P. E. , Jackson, S. K. , Henry, R. R. , Mathieu‐Costello, O. , McCord, J. M. , & Richardson, R. S. (2007). Electron paramagnetic spectroscopic evidence of exercise‐induced free radical accumulation in human skeletal muscle. Free Radical Research, 41(2), 182–190. 17364944 10.1080/10715760601028867

[eph70314-bib-0005] Bailey, D. M. , Rasmussen, P. , Evans, K. A. , Bohm, A. M. , Zaar, M. , Nielsen, H. B. , Brassard, P. , Nordsborg, N. B. , Homann, P. H. , Raven, P. B. , McEneny, J. , Young, I. S. , McCord, J. M. , & Secher, N. H. (2018). Hypoxia compounds exercise‐induced free radical formation in humans; partitioning contributions from the cerebral and femoral circulation. Free Radical Biology and Medicine, 124, 104–113. 29859345 10.1016/j.freeradbiomed.2018.05.090

[eph70314-bib-0006] Bailey, D. M. , Rasmussen, P. , Overgaard, M. , Evans, K. A. , Bohm, A. M. , Seifert, T. , Brassard, P. , Zaar, M. , Nielsen, H. B. , Raven, P. B. , & Secher, N. H. (2017). Nitrite and s‐nitrosohemoglobin exchange across the human cerebral and femoral circulation: Relationship to basal and exercise blood flow responses to hypoxia. Circulation, 135(2), 166–176. 27881556 10.1161/CIRCULATIONAHA.116.024226

[eph70314-bib-0007] Blaber, A. P. , Bondar, R. L. , Stein, F. , Dunphy, P. T. , Moradshahi, P. , Kassam, M. S. , & Freeman, R. (1997). Transfer function analysis of cerebral autoregulation dynamics in autonomic failure patients. Stroke, 28(9), 1686–1692. 9303010 10.1161/01.str.28.9.1686

[eph70314-bib-0008] Burley, C. V. , Bailey, D. M. , Marley, C. J. , & Lucas, S. J. E. (2016). Brain train to combat brain drain; focus on exercise strategies that optimize neuroprotection. Experimental Physiology, 101(9), 1178–1184. 27443587 10.1113/EP085672

[eph70314-bib-0009] Calverley, T A. , Ogoh, S. , Marley, C. J. , Steggall, M. , Marchi, N. , Brassard, P. , Lucas, S. J. E. , Cotter, J D. , Roig, M. , Ainslie, P N. , Wisløff, U. , & Bailey, D M. (2020). HIITing the brain with exercise: Mechanisms, consequences and practical recommendations. The Journal of Physiology, 598(13), 2513–2530. 32347544 10.1113/JP275021

[eph70314-bib-0010] Davison, G. W. , George, L. , Jackson, S. K. , Young, I. S. , Davies, B. , Bailey, D. M. , Peters, J. R. , & Ashton, T. (2002). Exercise, free radicals, and lipid peroxidation in type 1 diabetes mellitus. Free Radical Biology and Medicine, 33(11), 1543–1551. 12446212 10.1016/s0891-5849(02)01090-0

[eph70314-bib-0011] Erickson, K. I. , Voss, M. W. , Prakash, R. S. , Basak, C. , Szabo, A. , Chaddock, L. , Kim, J. S. , Heo, S. , Alves, H. , White, S. M. , Wojcicki, T. R. , Mailey, E. , Vieira, V. J. , Martin, S. A. , Pence, B. D. , Woods, J. A. , McAuley, E. , & Kramer, A. F. (2011). Exercise training increases size of hippocampus and improves memory. Proceedings of the National Academy of Sciences, USA, 108(7), 3017–3022. 10.1073/pnas.1015950108 PMC3041121 21282661

[eph70314-bib-0012] Febbraio, M. A. , & Pedersen, B. K. (2026). Exercise as a therapeutic intervention for long‐lasting and chronic diseases. Cell Metabolism, 38(5), 844–862. 41875883 10.1016/j.cmet.2026.02.017

[eph70314-bib-0013] Fogarty, M. C. , Hughes, C. M. , Burke, G. , Brown, J. C. , Trinick, T. R. , Duly, E. , Bailey, D. M. , & Davison, G. W. (2011). Exercise‐induced lipid peroxidation: Implications for deoxyribonucleic acid damage and systemic free radical generation. Environmental and Molecular Mutagenesis, 52(1), 35–42. 20839226 10.1002/em.20572

[eph70314-bib-0014] Globus, M. , Melamed, E. , Keren, A. , Tzivoni, D. , Granot, C. , Lavy, S. , & Stern, S. (1983). Effect of exercise on cerebral circulation. Journal of Cerebral Blood Flow & Metabolism, 3(3), 287–290. 6409908 10.1038/jcbfm.1983.43

[eph70314-bib-0015] Gomez‐Pinilla, F. , & Hillman, C. (2013). The influence of exercise on cognitive abilities. Comprehensive Physiology, 3(1), 403–428. 23720292 10.1002/cphy.c110063 PMC3951958

[eph70314-bib-0016] Heistad, D. D , & Kontos, H. A . (1983). Cerebral circulation. In J. T. Shepherd , & F. M. Abboud (Eds.), Handbook of physiology: The cardiovascular system. peripheral circulation and organ blood flow (Vol., 3, pp. 137–182). American Physiological Society.

[eph70314-bib-0017] Kety, S. S. , & Schmidt, C. F. (1946). The Effects of Active and Passive Hyperventilation on Cerebral Blood Flow, Cerebral Oxygen Consumption, Cardiac Output, and Blood Pressure of Normal Young Men. Journal of Clinical Investigation, 25(1), 107–119. 21016304

[eph70314-bib-0018] Lassen, N. A. (1959). Cerebral blood flow and oxygen consumption in man. Physiological Reviews, 39(2), 183–238. 13645234 10.1152/physrev.1959.39.2.183

[eph70314-bib-0019] Lucas, S. J. , Tzeng, Y. C. , Galvin, S. D. , Thomas, K. N. , Ogoh, S. , & Ainslie, P. N. (2010). Influence of changes in blood pressure on cerebral perfusion and oxygenation. Hypertension, 55(3), 698–705. 20083726 10.1161/HYPERTENSIONAHA.109.146290

[eph70314-bib-0020] Madsen, P. L. , Sperling, B. K. , Warming, T. , Schmidt, J. F. , Secher, N. H. , Wildschiodtz, G. , Holm, S. , & Lassen, N. A. (1993). Middle cerebral artery blood velocity and cerebral blood flow and O2 uptake during dynamic exercise. Journal of Applied Physiology, 74(1), 245–250. 8444699 10.1152/jappl.1993.74.1.245

[eph70314-bib-0021] Mandolesi, L. , Polverino, A. , Montuori, S. , Foti, F. , Ferraioli, G. , Sorrentino, P. , & Sorrentino, G. (2018). Effects of physical exercise on cognitive functioning and wellbeing: Biological and psychological benefits. Frontiers in Psychology, 9, 509. 29755380 10.3389/fpsyg.2018.00509 PMC5934999

[eph70314-bib-0022] Matura, S. , Fleckenstein, J. , Deichmann, R. , Engeroff, T. , Füzéki, E. , Hattingen, E. , Hellweg, R. , Lienerth, B. , Pilatus, U. , Schwarz, S. , Tesky, V. A. , Vogt, L. , Banzer, W. , & Pantel, J. (2017). Effects of aerobic exercise on brain metabolism and grey matter volume in older adults: Results of the randomised controlled SMART trial. Translational Psychiatry, 7(7), e1172. 28934191 10.1038/tp.2017.135 PMC5538117

[eph70314-bib-0023] Obrist, W. D. , Jr. Thompson, H. K. , King, C. H. , & Wang, H. S. (1967). Determination of regional cerebral blood flow by inhalation of 133‐Xenon. Circulation Research, 20(1), 124–135. 6017706 10.1161/01.res.20.1.124

[eph70314-bib-0024] Ogoh, S. (2008). Autonomic control of cerebral circulation: Exercise. Medicine & Science in Sports & Exercise, 40(12), 2046–2054. 18981945 10.1249/MSS.0b013e318180bc6f

[eph70314-bib-0025] Ogoh, S. (2017). Relationship between cognitive function and regulation of cerebral blood flow. The Journal of Physiological Sciences, 67(3), 345–351. 28155036 10.1007/s12576-017-0525-0 PMC10717011

[eph70314-bib-0026] Ogoh, S. (2019). Interaction between the respiratory system and cerebral blood flow regulation. Journal of Applied Physiology, 127(5), 1197–1205. 30920887 10.1152/japplphysiol.00057.2019

[eph70314-bib-0027] Ogoh, S. (2025). Cardiac output‐mediated regulation of cerebral blood flow during exercise: Clinical perspectives on the indirect impact of muscle metaboreflex. Experimental Physiology, 110(5), 686–693. 38500291 10.1113/EP091591 PMC12053890

[eph70314-bib-0028] Ogoh, S. , & Ainslie, P. N. (2009a). Cerebral blood flow during exercise: Mechanisms of regulation. Journal of Applied Physiology, 107(5), 1370–1380. 19729591 10.1152/japplphysiol.00573.2009

[eph70314-bib-0029] Ogoh, S. , & Ainslie, P. N. (2009b). Regulatory mechanisms of cerebral blood flow during exercise: New concepts. Exercise and Sport Sciences Reviews, 37(3), 123–129. 19550203 10.1097/JES.0b013e3181aa64d7

[eph70314-bib-0030] Ogoh, S. , Nakata, H. , Kubo, H. , & Shibasaki, M. (2024). Do changes in cerebral blood flow modulate the amplitudes of P300 during cognitive task? Journal of Applied Physiology, 137(5), 1106–1116. 39262339 10.1152/japplphysiol.00351.2024

[eph70314-bib-0031] Ogoh, S. , Sato, K. , Okazaki, K. , Miyamoto, T. , Hirasawa, A. , Morimoto, K. , & Shibasaki, M. (2013). Blood flow distribution during heat stress: Cerebral and systemic blood flow. Journal of Cerebral Blood Flow & Metabolism, 33(12), 1915–1920. 23942362 10.1038/jcbfm.2013.149 PMC3851900

[eph70314-bib-0032] Ogoh, S. , & Tarumi, T. (2019). Cerebral blood flow regulation and cognitive function: A role of arterial baroreflex function. The Journal of Physiological Sciences, 69(6), 813–823. 31444691 10.1007/s12576-019-00704-6 PMC10717347

[eph70314-bib-0033] Ogoh, S. , Tsukamoto, H. , Hirasawa, A. , Hasegawa, H. , Hirose, N. , & Hashimoto, T. (2014). The effect of changes in cerebral blood flow on cognitive function during exercise. Physiological Reports, 2(9), e12163. 25263210 10.14814/phy2.12163 PMC4270220

[eph70314-bib-0034] Ogoh, S. , Washio, T. , Stacey, B. S. , Tsukamoto, H. , Iannetelli, A. , Owens, T. S. , Calverley, T. A. , Fall, L. , Marley, C. J. , & Bailey, D. M. (2022). Effects of continuous hypoxia on flow‐mediated dilation in the cerebral and systemic circulation: On the regulatory significance of shear rate phenotype. The Journal of Physiological Sciences, 72(1), 16. 35858836 10.1186/s12576-022-00841-5 PMC10717978

[eph70314-bib-0043] Rowell, L. B. (1986). Human circulation: Regulation during physical stress . Oxford University Press.

[eph70314-bib-0035] Sallis, R. E. (2009). Exercise is medicine and physicians need to prescribe it! British Journal of Sports Medicine, 43(1), 3–4. 18971243 10.1136/bjsm.2008.054825

[eph70314-bib-0036] Sato, K. , Oba, N. , Washio, T. , Sasaki, H. , Oue, A. , Otsuki, A. , Sadamoto, T. , & Ogoh, S. (2017). Relationship between cerebral arterial inflow and venous outflow during dynamic supine exercise. Physiological Reports, 5(12), e13292. 28663325 10.14814/phy2.13292 PMC5492200

[eph70314-bib-0037] Scheinberg, P. , Blackburn, L. I. , Rich, M. , & Saslaw, M. (1954). Effects of vigorous physical exercise on cerebral circulation and metabolism. The American Journal of Medicine, 16(4), 549–554. 13148198 10.1016/0002-9343(54)90371-x

[eph70314-bib-0038] Shibasaki, M. , Sato, K. , Hirasawa, A. , Sadamoto, T. , Crandall, C. G. , & Ogoh, S. (2020). An assessment of hypercapnia‐induced elevations in regional cerebral perfusion during combined orthostatic and heat stresses. The Journal of Physiological Sciences, 70(1), 25. 32366213 10.1186/s12576-020-00751-4 PMC8006159

[eph70314-bib-0039] Shipley, R. E. , & Study, R. S. (1951). Changes in renal blood flow, extraction of inulin, glomerular filtration rate, tissue pressure and urine flow with acute alterations of renal artery blood pressure. American Journal of Physiology, 167(3), 676–688. 14903093 10.1152/ajplegacy.1951.167.3.676

[eph70314-bib-0040] Stacey, B. S. , Marley, C. J. , Tsukamoto, H. , Dawkins, T. G. , Owens, T. S. , Calverley, T. A. , Fall, L. , Iannetelli, A. , Lewis, I. , Coulson, J. M. , Stembridge, M. , & Bailey, D. M. (2025). Phosphodiesterase inhibition restores hypoxia‐induced cerebrovascular dysfunction subsequent to improved systemic redox homeostasis: A randomized, double‐blind, placebo‐controlled crossover study. Journal of Cerebral Blood Flow & Metabolism, 45(7), 1343–1356. 39862172 10.1177/0271678X251313747 PMC11765346

[eph70314-bib-0041] Tyndall, A. V. , Clark, C. M. , Anderson, T. J. , Hogan, D. B. , Hill, M. D. , Longman, R. S. , & Poulin, M. J. (2018). Protective effects of exercise on cognition and brain health in older adults. Exercise and Sport Sciences Reviews, 46(4), 215–223. 30001269 10.1249/JES.0000000000000161

[eph70314-bib-0042] Zhang, R. , Zuckerman, J. H. , Giller, C. A. , & Levine, B. D. (1998). Transfer function analysis of dynamic cerebral autoregulation in humans. American Journal of Physiology, 274(1 Pt 2), H233–H241. 9458872 10.1152/ajpheart.1998.274.1.h233

